# Dataset for forensic analysis of B-tree file system

**DOI:** 10.1016/j.dib.2018.04.100

**Published:** 2018-05-03

**Authors:** Mohamad Ahtisham Wani, Wasim Ahmad Bhat

**Affiliations:** Department of Computer Sciences, University of Kashmir, India

## Abstract

Since B-tree file system (Btrfs) is set to become *de facto* standard file system on Linux (and Linux based) operating systems, Btrfs dataset for forensic analysis is of great interest and immense value to forensic community. This article presents a novel dataset for forensic analysis of Btrfs that was collected using a proposed data-recovery procedure. The dataset identifies various generalized and common file system layouts and operations, specific node-balancing mechanisms triggered, logical addresses of various data structures, on-disk records, recovered-data as directory entries and *extent* data from leaf and internal nodes, and percentage of data recovered.

**Specifications Table**

**Table:** 

Subject area	*Computer Science*
More specific subject area	*Computer Forensics, File System Forensic Analysis*
Type of data	*Table, Figure*
How data was acquired	*Data was extracted and recorded using the proposed data-recovery procedure.*
Data format	*Raw*
Experimental factors	*None*
Experimental features	*Data recovery using orphan-item analysis was proposed and validated through post-process identification and extraction.*
Data source location	*University of Kashmir, India*
Data accessibility	*Data is available within this article.*

**Value of the data**

• Forensic analysis of file systems generally relies on data recovery to yield credible and conclusive investigation [1], [2], [3]. Therefore, a dataset that describes changes incurred by a file system during data deletion and/or modification is of immense value to forensic community.
• B-tree file system (Btrfs) is the most advanced, multi-platform, and scalable file system that is set to become the default Linux file system [4]. Therefore, forensic analysis of Btrfs is imperative, and this dataset serves the stepping-stone.
• The dataset was captured by employing a proposed data-recovery procedure for Btrfs. The dataset captures all aspects of the data on the file system which includes logical layouts, operations performed, on-disk records, logical addresses, node-balancing mechanisms, recovered-data ratios, and so on.
• The dataset shows the significance of node-balancing and Copy-on-write (COW) model of Btrfs in data recovery. Thus, the forensic value of the dataset is eminent.
• Academicians, researchers, digital forensic investigators, and developers can exploit the dataset to get valuable insights [5] into the behavior of Btrfs.

## 1 Data

### 1.1 Rationale

Linux operating system is most commonly and widely used operating system across all platforms and domains. Over a period of more than two decades, Linux file systems have evolved significantly. Ext4 is one of the most popular and last in the line of Linux extended file systems. It has been the default choice for most of the Linux distributions in recent years. Although it was a big improvement over its predecessors, its aging code base is unable to support evolving demands of data integrity, deduplication and survivability, disk diversity, fault isolation, light weight snapshots and clones, checksums for reliability, and online compression and defragmentation for performance. Basically, the idea behind Ext4 design was to create a stop-gap solution until a stable version of Btrfs was ready [6]. Btrfs addresses these challenges of reliability, scalability and performance by providing simple administration, end-to-end data integrity, and immense scalability without loss of performance. Therefore, Btrfs delivers what Ext4 fails to, i.e., maintaining an even performance across sensitive, intense and diverse workloads managed by Linux operating systems, be it smartphones, enterprise production servers, or modern super computers. With such a diverse and sensitive workload to shoulder, Btrfs is at the spotlight of hackers, malicious-code writers and cyber criminals. All file systems are vulnerable to breach [5], [7], [8], and Btrfs is not an exception. Forensic investigators rely on file system forensic artefacts to analyze such breaches [9], recreate digital crime-scene [10], possibly unveil intruder intentions, and recover deleted or modified data [11]. Since file systems vary greatly in design, so do the forensic artefacts they yield and the data-recovery procedures to harvest them. The data-recovery procedures yield forensic datasets that allow forensic investigators to analyse the behavior of file systems, identify forensically important data structures, devise mechanisms for their extraction, and determine the probability of finding digital evidences. Therefore, the forensic datasets are of great interest and immense value to forensic investigators. And, with the inevitable adoption of Btrfs across wide platforms and diverse workloads, forensic dataset of Btrfs is of greater interest and bigger value to forensic community.

### 1.2 Btrfs forensic dataset

**Fig. 1:**
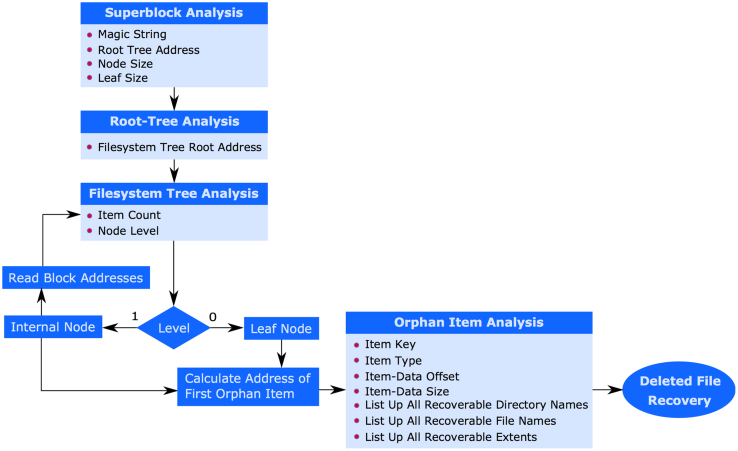
Proposed B-tree file system data-recovery procedure.

Based on the design of Btrfs and the state-change incurred by the file system during file and directory operations, we propose a 6-step data-recovery procedure for Btrfs as shown in Fig. 1.

The dataset generated by the proposed data-recovery procedure is shown in Table S1. The dataset identifies various generalized and common file system layouts and operations, specific node-balancing mechanisms triggered, logical addresses of various data structures, on-disk records, recovered-data as directory entries and *extent* data from leaf and internal nodes, and percentage of data recovered.

### 1.3 Dataset description

• “**Logical layout of the file system”** shows the logical layout of directories and files in the root directory of Btrfs volume. It should be noted that all regular files have an extension *‘.txt’* in their names, and no such suffix is added to directory names.
• “**Operations performed”** specifies the operation performed on files and/or directories of the file system.
• **“Node-balancing mechanism triggered”** indicates the balancing mechanism that is triggered if the performed operation unbalances the underlying file system B-trees.
• **“File system tree root address”** specifies the root address of the file system B-tree.
• **“Level”** indicates the *level* of the node. Its value can be either 1 (internal node) or 0 (leaf node).
• “**Items”** specifies the node's *item_count* which defines the number of records contained within the nodes. It is worth mentioning here that for Experiment No. 4, 6, 8, 15, 17, 22, 24, 26, the file system layout is empty (i.e. the root directory contains no apparent files or directories) but the “Items” column contains a non-zero value. This is because every directory of a file system always maintains two directories, i.e., current directory (denoted by .) and the parent directory (denoted by ..). In addition, the *root* directory also contains temporary directory entries (like*.Trash-000000004*,*.Trash-00000010a*, *expunged00000004*, *expunged0000010f*, *files*, *info*, and so on) along with their *inode* and index records. These entries are insignificant in data recovery, and hence “on-disk records” column has been put as *Not Important* for the above mentioned experiments.
• “**Block addresses”** specifies block addresses contained within internal nodes that point to other internal or leaf nodes.
• **“On-disk records”** shows on-disk file system metadata corresponding to files and directories present in the current file system layout. The directories are represented by a single record containing two *object_ids*. The suffixed *object_id* identifies the directory itself while as the prefixed *object_id* identifies its parent directory. In contrast, regular-files are represented by two records – directory entry and extent data. The directory entry record is same as that for a directory while as extent data record contains information on actual file content and contains the *object_id* of the file as its suffix. *object_ids* are critical in understanding the behavior of B-tree file system, particularly in case of metadata operations (like move, rename, modify, etc.) where changes occur at metadata level. Besides, in order to make a clear distinction between a “Directory entry” records of directory and file, regular-files are named with a *.txt* extension.
• **“Recovered data (from leaf-nodes)”** shows the file system metadata and data recovered from leaf nodes of the file system in the form of *Orphan_Items*. These *items* exist beyond the *item_count* value of the node and contain data remnants of previously deleted data. The data is further classified into **“Directory entries”** and **“Extent data”** in order to differentiate between directory entries and file content respectively.
• **“Recovered data (from internal-nodes)”** shows the file system metadata and data recovered from internal nodes of the file system in the form of *Orphan_Items*. The data is further classified into **“Directory entries”** and **“Extent data”** in order to differentiate between directory entries and file content respectively.
• **“Percentage of data recovered”** is the percentage of data recovered from internal and leaf nodes. Unlike directories where recovery of directory entries is enough, regular-file recovery demands recovery of file content stored in various *extent* types along with their respective directory entries. Hence, regular-files are accounted for two entities when recovery ratio is calculated.

## 2 Experimental design, materials and methods

### 2.1 Platform and tools

The experiment employed a 64-bit Fedora Core 23 Linux operating system running kernel v4.2. Btrfs was introduced in Linux mainline kernel v2.6.29 in March 2009. Since then Btrfs support has matured through various subsequent Linux kernel releases with v4.15 released in January 2018 being the latest one. The proposed data-recovery procedure was implemented in C programming language.

### 2.2 Procedure

**Fig. 2:**
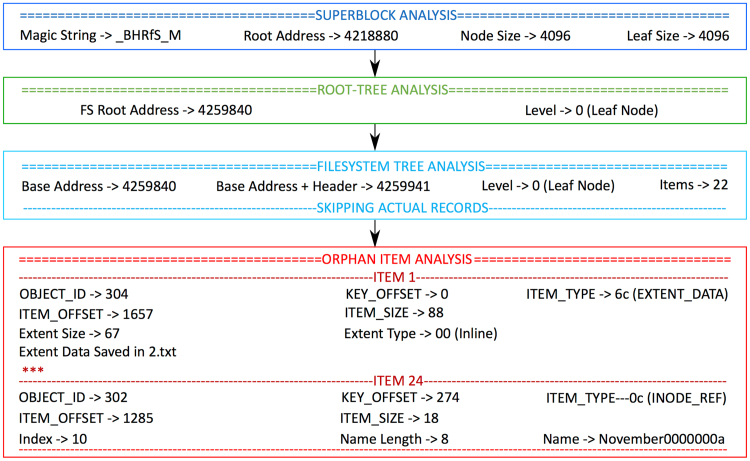
Output of different stages of the proposed data-recovery procedure for one of the Use cases.

The program traverses the file system B-tree, and parses its data structures for internal and leaf nodes. When a node is identified, the program analyzes the node for *Orphan-Items* and extracts data only from those *Orphan-Items* that contain valid data as per a pre-defined *valid-entry lookup* table. Fig. 2 shows the result of each step of the data-recovery procedure during one of the Use cases.

### 2.3 Use case description

The experiment comprised of carefully chosen Use-cases. The Use-cases were constructed keeping in view the following:

• It has been found that most of the directories in a file system are shallow and upto 4 levels deep [12], [13], [14]. Therefore, we simulated different file and directory organizations on the file system that reflects this observed common-layout.
• Node-balancing is at the core of Btrfs that decides the percentage of data recovered. Hence, such Use cases were also designed that guaranteed either redistribution or merging of nodes.
• In a fresh B-tree file system, atleast 4 regular files or directories are required for internal nodes to exist. This is because the space required for their metadata is large enough to guarantee overflow in the leaf block, which eventually results into splitting of leaf and creation of internal node in the process. This allows internal nodes to be evaluated for data recoverability.
• A file in Btrfs is stored either in *inline-extent* or *regular–extent* depending upon the file size. Specific Use cases were designed that contained both small and large files so that the relationship between file size and node-balancing can be determined, and *extent-types* can be evaluated for data recoverability.
• For metadata operations, no specific file system layout is required as the changes resulting from metadata operations are made in-place. Thus, the percentage of data-recovered in such cases is neither affected by the layout nor do the resulting changes affect the layout.

## Footnotes

Appendix A Supplementary data associated with this article can be found in the online version at 10.1016/j.dib.2018.04.100.
