# Supplementary material for: Dataset for forensic analysis of B-tree file system
Source: Data Brief. 2018 May 3;18:2013–8. doi: 10.1016/j.dib.2018.04.100 (PMC5998747; doi:10.1016/j.dib.2018.04.100)
Supplement: Supplementary file 2 — Supplementary material [file mmc2.docx]

| S.No. | Logical layout of file system | Operations performed | Node-balancing mechanism triggered | File system tree root address | Level | Items | Block addresses | Level | Items | On-disk records | | Recovered data (from leaf nodes) | | Recovered data  (from internal nodes) | | Percentage of data recovered |
| --- | --- | --- | --- | --- | --- | --- | --- | --- | --- | --- | --- | --- | --- | --- | --- | --- |
|  |  |  |  |  |  |  |  |  |  | Directory entries | Extent data | Orphan items | | Orphan items | |  |
|  |  |  |  |  |  |  |  |  |  |  |  | Directory entries | Extent data | Directory entries | Extent data |  |
| 1 |  | Add Alpha, Bravo, Charlie, Delta.txt, Echo.txt and Foxtrot.txt | Not Applicable | 4194304 | 1 | 2 | 4202496 | 0 | 19 | 256-Alpha-278, 256-Bravo-281, 256-Echo.txt-280, 256-Delta.txt-277 | Delta.txt-277, Echo.txt-280 | Not Possible | Not Possible | Not Possible | Not Possible | Not Applicable |
|  |  |  |  |  |  |  | 4198400 | 0 | 16 | 256-Charlie-282, 256-Foxtrot.txt-279 | Foxtrot.txt-279 |  |  |  |  |  |
| 2 |  | Delete Alpha and Delta.txt | Redistribution | 4206592 | 1 | 2 | 4222976 | 0 | 26 | 256-Bravo-281, 256-Echo.txt-280 | Echo.txt-280 | 256-Alpha-278 | Nothing Found | Nothing Found | Nothing Found | 33.3% |
|  |  |  |  |  |  |  | 4214784 | 0 | 18 | 256-Charlie-282, 256-Foxtrot.txt-279 | Foxtrot.txt-279 | Nothing Found | Foxtrot.txt-279 |  |  |  |
| 3 |  | Delete Foxtrot.txt | Not Applicable | 6160384 | 1 | 2 | 4194304 | 0 | 26 | 256-Bravo-281, 256-Echo.txt-280 | Echo.txt-280 | 256-Alpha-278 | Nothing Found | Nothing Found | Nothing Found | 50% |
|  |  |  |  |  |  |  | 4202496 | 0 | 14 | 256-Charlie-282 | Not Available | Nothing Found | Foxtrot.txt-279 |  |  |  |
| 4 |  | Delete All Data | Merging | 4198400 | 0 | 22 | Not Applicable | Not Applicable | Not Applicable | Not Important | Not Important | 256-Alpha-278, 256-Echo.txt-280 | Nothing Found | Nothing Found | Nothing Found | 25% |
| 5 |  | Add Installed.txt | Not Applicable | 6160384 | 0 | 23 | Not Applicable | Not Applicable | Not Applicable | 256-Installed.txt-283 | Installed.txt-283 | 256-Alpha-278, 256-Echo.txt-280 | Nothing Found | Nothing Found | Nothing Found | Not Applicable |
| 6 |  | Delete Installed.txt | Not Applicable | 4202496 | 0 | 22 | Not Applicable | Not Applicable | Not Applicable | Not Important | Not Important | 256-Alpha-278, 256-Installed.txt-283 | Installed.txt-283, Foxtrot.txt-279, Echo.txt-280 | Nothing Found | Nothing Found | 100% |
| 7 |  | Add Golf and Houston.txt | Not Applicable | 4202496 | 0 | 33 | Not Applicable | Not Applicable | Not Applicable | 256-Golf-285, 256-Houston.txt-284 | Houston.txt-284 | 256-Alpha-278, 256-Installed.txt-283 | Nothing Found | Nothing Found | Nothing Found | Not Applicable |
| 8 |  | Delete Golf and Houston.txt | Not Applicable | 4194304 | 0 | 22 | Not Applicable | Not Applicable | Not Applicable | Not Important | Not Important | 256-Houston.txt-284, 256-Golf-285 | Houston.txt-284 | Nothing Found | Nothing Found | 100% |
| 9 |  | Add Indigo | Not Applicable | 4198400 | 0 | 27 | Not Applicable | Not Applicable | Not Applicable | 256-Indigo-289 | Not Important | 256-Houston.txt-284, 256-Golf-285 | Houston.txt-284 | Nothing Found | Nothing Found | Not Applicable |
| 10 |  | Add Kali.txt | Not Applicable | 4194304 | 0 | 33 | Not Applicable | Not Applicable | Not Applicable | 256-Indigo-289, 256-Kali.txt-290 | Kali.txt-290 | 256-Houston.txt-284, 256-Golf-285 | Houston.txt-284 | Nothing Found | Nothing Found | Not Applicable |
| 11 |  | Add Juliet | Not Applicable | 4198400 | 0 | 38 | Not Applicable | Not Applicable | Not Applicable | 256-Indigo-289, 256-Kali.txt-290,  256-Juliet-291 | Kali.txt-290 | 256-Golf-285 | Nothing Found | Nothing Found | Nothing Found | Not Applicable |
| 12 |  | Add Lima.txt | Redistribution | 4214784 | 1 | 2 | 4194304 | 0 | 22 | 256-Indigo-289, 256-Kali.txt-290,  256-Juliet-291, 256-Lima.txt-292 | Not Available | 256-Indigo-289, 256-Kali.txt-290,  256-Juliet-291, 256-Lima.txt-292 | Kali.txt-290 | Nothing Found | Nothing Found | Not Applicable |
|  |  |  |  |  |  |  | 4222976 | 0 | 22 | Not Available | Kali.txt-290, Lima.txt-292 | Nothing Found | Nothing Found |  |  |  |
| 13 |  | Delete Juliet | Redistribution | 4198400 | 1 | 2 | 4243456 | 0 | 20 | 256-Indigo-289, 256-Kali.txt-290,  256-Lima.txt-292 | Not Available | 256-Indigo-289, 256-Kali.txt-290,  256-Juliet-291, 256-Lima.txt-292 | Kali.txt-290 | Nothing Found | Nothing Found | 100% |
|  |  |  |  |  |  |  | 4202496 | 0 | 15 | Not Available | Kali.txt-290, Lima.txt-292 | 256-Juliet-291, | Lima.txt-292 |  |  |  |
| 14 |  | Delete Lima.txt | Not Applicable | 4194304 | 1 | 2 | 4222976 | 0 | 18 | 256-Indigo-289, 256-Kali.txt-290 | Not Available | 256-Indigo-289, 256-Kali.txt-290,  256-Juliet-291, 256-Lima.txt-292 | Kali.txt-290 | Nothing Found | Nothing Found | 100% |
|  |  |  |  |  |  |  | 4214784 | 0 | 15 | Not Available | Kali.txt-290 | Nothing Found | Lima.txt-292 |  |  |  |
| 15 |  | Delete All Data | Merging | 4243456 | 0 | 22 | Not Applicable | Not Applicable | Not Applicable | Not Important | Not Important | 256-Indigo-289, 256-Kali.txt-290,  256-Juliet-291, 256-Lima.txt-292 | Kali.txt-290 | Nothing Found | Nothing Found | 100% |
| 16 |  | Add Mike, November, Oscar, Papa.txt, Quebec.txt, and Romeo.txt | Redistribution | 4214784 | 1 | 2 | 4194304 | 0 | 29 | 256-November-302, 256-Oscar-303, 256-Papa.txt-304, 256-Quebec.txt-305 | Papa.txt-304 | 256-November-302, 256-Oscar-303, 256-Papa.txt-304, 256-Quebec.txt-305 | Nothing Found | Nothing Found | Nothing Found | Not Applicable |
|  |  |  |  |  |  |  | 4218880 | 0 | 23 | 256-Mike-301, 256-Romeo.txt-306 | Quebec.txt-305, Romeo.txt-306 | Nothing Found | Nothing Found |  |  |  |
| 17 |  | Delete All Data | Merging | 4259840 | 0 | 22 | Not Applicable | Not Applicable | Not Applicable | Not Important | Not Important | 256-November-302, 256-Mike-301, 256-Papa.txt-304 | Papa.txt-304 | Nothing Found | Nothing Found | 44.4% |
| 18 |  | Add Tango, Sierra, Uniform, Victor.txt, Whiskey, X-Ray.txt, and Zebra.txt | Redistribution | 4198400 | 1 | 2 | 4218880 | 0 | 40 | 256-Tango-324, 256-Sierra-319, 319-Whiskey-322, 319-Victor.txt-326, 319- Uniform-320, 319-X-Ray.txt-327 | Not Available | Nothing Found | Nothing Found | Nothing Found | Nothing Found | Not Applicable |
|  |  |  |  |  |  |  | 4222976 | 0 | 20 | 324-Zebra.txt-325 | Victor.txt-326, X-Ray.txt-327, Zebra.txt-325 | Nothing Found | Zebra.txt-325, Victor.txt-326 |  |  |  |
| 19 |  | Delete Zebra.txt | Not Applicable | 4194304 | 1 | 2 | 4227072 | 0 | 40 | 256-Tango324, 256-Sierra-319, 319-Whiskey-322, 319-Victor.txt-326, 319- Uniform-320, 319-X-Ray-327 | Not Available | Nothing Found | Nothing Found | 256-Tango-324, 256-Sierra-319, 319-Victor.txt-326, 319-X-Ray-327, 319-Uniform-320 | Nothing Found | 100% |
|  |  |  |  |  |  |  | 4202496 | 0 | 14 | Not Available | Victor.txt-326, X-Ray.txt-327, | 324-Zebra.txt-325 | X-Ray.txt-327, Zebra.txt-325, Zebra.txt-330, Victor.txt-326 |  |  |  |
| 20 |  | Delete X-Ray.txt | Redistribution | 4194304 | 1 | 2 | 4231168 | 0 | 43 | 256-Tango-324, 256-Sierra-319, 319-Whiskey -322, 319-Victor.txt-326, 319- Uniform-320 | Not Available | 256-Tango-324 | Nothing Found | 256-Romeo.txt-306, 256-Quebec.txt-305, 256-Papa.txt-304, 256-Oscar-303 | Nothing Found | 100% |
|  |  |  |  |  |  |  | 4202496 | 0 | 5 | Not Available | Victor.txt-326 | 324-Zebra.txt-325, 319-X-Ray.txt-327 | Victor.txt-326, X-ray.txt-327 |  |  |  |
| 21 |  | Add Yankee.txt (4K) | Not Applicable | 4194304 | 1 | 2 | 4259840 | 0 | 43 | 256-Tango-324, 256-Sierra-319, 319-Whiskey-322, 319-Victor.txt-326, 319- Uniform-320 | Not Available | 256-Tango-324 | Nothing Found | 256-Romeo.txt-306, 256-Quebec.txt-305, 256-Papa.txt-304, 256-Oscar-303 | Nothing Found | Not Applicable |
|  |  |  |  |  |  |  | 4227072 | 0 | 11 | 324-Yankee.txt-333 | Victor.txt-326(I), Yankee.txt-333(R) | 324-Zebra.txt-325, X-Ray.txt | X-Ray.txt-327, Yankee.txt-333, Kali.txt-325, Victor.txt-326 |  |  |  |
| 22 |  | Delete All Data | Merging | 4214784 | 0 | 22 | Not Applicable | Not Applicable | Not Applicable | Not Important | Not Important | 324-Yankee.txt-333, Tango-324 | Victor.txt-326, Yankee.txt-333 | Nothing Found | Nothing Found | 50% |
| 23 |  | Add Alpha, Beta, Charlie, Delta.txt, Echo.txt, Foxtrot.txt, and Golf.txt | Redistribution | 4194304 | 1 | 2 | 4198400 | 0 | 24 | 256-Beta-285, 256-Alpha-280, 285-Golf.txt-286 | Golf.txt-286 | 256-Alpha-280, 280-Delta.txt-282, 280-Echo-283, 280-Charlie-281 | Nothing Found | Nothing Found | Nothing Found | Not Applicable |
|  |  |  |  |  |  |  | 4231168 | 0 | 36 | 280-Foxtrot.txt-284, 280-Delta.txt-282, 280-Echo.txt-283, 280-Charlie-281 | Delta.txt-282, Echo.txt-283, Foxtrot.txt-284 | 256-Tango-341, 324-Yankee.txt-342 | Nothing Found |  |  |  |
| 24 |  | Delete All Data | Merging | 4222976 | 0 | 22 | Not Applicable | Not Applicable | Not Applicable | Not Important | Not Important | 280-Charlie-281, 280-Delta.txt-282, 280-Echo-283, 280-Foxtrot.txt-284 | Foxtrot.txt-284 | Nothing Found | Nothing Found | 45.4% |
| 25 |  | Add T2, Houston, Indigo, Jake, Kali.txt, Oscar.txt, Lima, Mike.txt, and Papa.txt | Redistribution | 4198400 | 1 | 2 | 4194304 | 0 | 32 | 256-T2-291, 291-Houston-292, 291-Indigo-298, , 293-Lima-294 | Not Available | 291-Jake-293, 293-Lima-294, 294-Papa.txt-295 | Nothing Found | 256-Alpha-280, 256-Beta-285, 280-Delta.txt-282, 280-Echo-283, 280-Charlie-281 | Nothing Found | Not Applicable |
|  |  |  |  |  |  |  | 4202496 | 0 | 39 | 291-Jake-293, 292-Kali.txt-297, 292-Mike.txt-296, 298-Oscar-299, 294-Papa.txt-295 | Papa.txt-295, Mike.txt-296, Kali.txt-297, Oscar.txt-299 | Nothing Found | Oscar.txt-299 |  |  |  |
| 26 |  | Delete All Data | Merging | 4251648 | 0 | 22 | Not Applicable | Not Applicable | Not Applicable | Not Important | Not Important | 291-Houston-292, 291-Indigo-298, 298-Oscar.txt-299 | Oscar.txt-299 | Nothing Found | Nothing Found | 30.7% |
| 27 |  | Add T3, Quebec, Romeo, Sierra, Tango.txt, Uniform, Victor.txt, Whiskey.txt, X-Ray.txt, Yankee, and Zebra.txt | Redistribution | 4206592 | 1 | 2 | 4194304 | 0 | 41 | 256-T3-302, 302-Quebec-303, 302-Romeo-308, 302-Whiskey.txt-312, 303-Tango.txt-307, 303-Sierra-304 | Not Available | 304-Uniform-305 | Nothing Found | 292-Kali.txt-297, 292-Jake-293, 293-Lima-294, 293-Mike.txt-296, 294-Papa.txt-295, Indigo-298, 298-Oscar-299 | Zebra.txt-325, Mike.txt-296, Kali.txt-297, Oscar.txt-299 | Not Applicable |
|  |  |  |  |  |  |  | 4214784 | 0 | 41 | 304-Victor.txt-306, 304-Uniform-305, 308-Yankee-309, 308-Zebra-311, 309-X-Ray-310 | Victor.txt-306, Tango.txt-307, X-Ray.txt-310, Zebra.txt-311, Whiskey.txt-312 | Nothing Found | Nothing Found |  |  |  |
| 28 |  | Rename Zebra.txt to Zebbra.txt | Not Applicable | 4198400 | 1 | 2 | 4231168 | 0 | 41 | 256-T3-302, 302-Quebec-303, 302-Romeo-308, 302-Whiskey.txt-312, 303-Tango.txt-307, 303-Sierra-304 | Not Available | 304-Uniform-305 | Nothing Found | 292-Kali.txt-297, 292-Jake-293, 293-Lima-294, 293-Mike.txt-296, 294-Papa.txt-295, Indigo-298, 298-Oscar-299 | Zebra.txt-325, Mike.txt-296, Kali.txt-297, Oscar.txt-299 | Not Applicable |
|  |  |  |  |  |  |  | 4202496 | 0 | 41 | 304-Victor.txt-306, 304-Uniform-305, 308-Yankee-309, 308-Zebbra-311, 309-X-Ray-310 | Victor.txt-306, Tango.txt-307, X-Ray.txt-310, Zebbra.txt-311, Whiskey.txt-312 | Nothing Found | Whiskey.txt-312 |  |  |  |
| 29 |  | Rename Tango.txt to Tngoo.txt | Not Applicable | 4194304 | 1 | 2 | 4214784 | 0 | 41 | 256-T3-302, 302-Quebec-303, 302-Romeo-308, 302-Whiskey.txt-312, 303-Tngoo.txt-307, 303-Sierra-304 | Not Available | Nothing Found | Nothing Found | 292-Kali.txt-297, 292-Jake-293, 293-Lima-294, 293-Mike.txt-296, 294-Papa.txt-295, Indigo-298, 298-Oscar-299 | Zebra.txt-325, Mike.txt-296, Kali.txt-297, Oscar.txt-299 | Not Applicable |
|  |  |  |  |  |  |  | 4206592 | 0 | 41 | 304-Victor.txt-306, 304-Uniform-305, 308-Yankee-309, 308-Zebbra-311, 309-X-Ray-310 | Victor.txt-306, Tngoo.txt-307, X-Ray.txt-310, Zebbra.txt-311, Whiskey.txt-312 | Nothing Found | Nothing Found |  |  |  |
| 30 |  | Modify DTS of T3 to "1988-10-31 00:00:00" | Not Applicable | 4198400 | 1 | 2 | 4202496 | 0 | 41 | 256-T3-302, 302-Quebec-303, 302-Romeo-308, 302-Whiskey.txt-312, 303-Tngoo.txt-307, 303-Sierra-304 | Not Available | Nothing Found | Nothing Found | 292-Kali.txt-297, 292-Jake-293, 293-Lima-294, 293-Mike.txt-296, 294-Papa.txt-295, Indigo-298, 298-Oscar-299 | Zebra.txt-325, Mike.txt-296, Kali.txt-297, Oscar.txt-299 | Not Applicable |
|  |  |  |  |  |  |  | 4206592 | 0 | 41 | 304-Victor.txt-306, 304-Uniform-305, 308-Yankee-309, 308-Zebbra-311, 309-X-Ray-310 | Victor.txt-306, Tngoo.txt-307, X-Ray.txt-310, Zebbra.txt-311, Whiskey.txt-312 | Nothing Found | Nothing Found |  |  |  |
| 31 |  | Modify Contents of file Victor.txt | Redistribution | 4198400 | 1 | 3 | 4227072 | 0 | 23 | 256-T3-302 | Not Available | 256-T3-302, 302-Quebec-303, 302-Romeo-308, 302-Whiskey.txt-312, 303-Tngoo.txt-307, 303-Sierra-304, 304-Victor.txt-306, 304-Uniform-305 | Nothing Found | 292-Kali.txt-297, 292-Jake-293, 293-Lima-294, 293-Mike.txt-296, 294-Papa.txt-295, Indigo-298, 298-Oscar-299 | Zebra.txt-325, Mike.txt-296, Kali.txt-297, Oscar.txt-299 | Not Applicable |
|  |  |  |  |  |  |  | 4268032 | 0 | 38 | 302-Quebec-303, 302-Romeo-308, 302-Whiskey.txt-312, 303-Tngoo.txt-307, 303-Sierra-304, 304-Victor.txt-318, 304-Uniform-305, 308-Yankee-309, 308-Zebbra-311 | Tngoo.txt-307 | 308-Zebbra.txt-311 | Nothing Found |  |  |  |
|  |  |  |  |  |  |  | 4231168 | 0 | 21 | 309-X-Ray-310 | Victor.txt-306, X-Ray.txt-310, Zebbra.txt-311, Whiskey.txt-312 | 302-Whiskey.txt-312 | Whiskey.txt-312, Victor.txt-318, Zebbra.txt-311 |  |  |  |
| 32 |  | Delete Uniform, Victor.txt, Romeo, Whiskey.txt, Yankee, X-Ray.txt, and Zebbra.txt | Merging | 4198400 | 1 | 2 | 4239360 | 0 | 23 | 256-T3-302 | Not Available | 303-Tngoo.txt-307, 303-Sierra-304, 304-Victor.txt-306, 304-Uniform-305 | Nothing Found | 292-Kali.txt-297, 292-Jake-293, 293-Lima-294, 293-Mike.txt-296, 294-Papa.txt-295, Indigo-298, 298-Oscar-299 | Zebra.txt-325, Mike.txt-296, Kali.txt-297, Oscar.txt-299 | 54.5% |
|  |  |  |  |  |  |  | 4251648 | 0 | 18 | 302-Quebec-303, 303-Tngoo.txt-307 | Tngoo.txt-307 | 308-Zebbra.txt-311, 308-Yankee-309, 302-Romeo-308 | Zebbra.txt-311 |  |  |  |
| 33 |  | Move Tngoo.txt to Root | Redistribution | 4194304 | 1 | 2 | 4214784 | 0 | 25 | 256-T3-302, 256-Tngoo.txt-307 | Not Available | 303-Tngoo.txt-307, 303-Sierra-304, 304-Victor.txt-306, 304-Uniform-305 | Nothing Found | 292-Kali.txt-297, 292-Jake-293, 293-Lima-294, 293-Mike.txt-296, 294-Papa.txt-295, Indigo-298, 298-Oscar-299 | Zebra.txt-325, Mike.txt-296, Kali.txt-297, Oscar.txt-299 | Not Applicable |
|  |  |  |  |  |  |  | 4202496 | 0 | 16 | 302-Quebec-303 | Tngoo.txt-307 | 308-Zebbra.txt-311, 308-Yankee-309, 302-Romeo-308 | Zebbra.txt-311 |  |  |  |
| 34 |  | Delete All Data | Merging | 4239360 | 0 | 25 | Not Applicable | Not Applicable | Not Applicable | Not Important | Not Important | 303-Sierra-304, 304-Victor.txt-306, 304-Uniform-305 | Nothing Found | Nothing Found | Nothing Found | 20% |

**Table 1:** Dataset generated by the proposed data-recovery procedure for B-tree file system.
